# Supplementary material for: The interactome of intact mitochondria by cross-linking mass spectrometry provides evidence for coexisting respiratory supercomplexes
Source: Mol Cell Proteomics. 2017 Dec 8;17(2):216–32. doi: 10.1074/mcp.RA117.000470 (PMC5795388; doi:10.1074/mcp.RA117.000470)
Supplement: Supplemental Data [file supp_RA117.000470_133922_0_supp_23978_fzffwf.zip › spectra_annotation/mito_spectra_annotation/244-1-5-1-4-1.pdf]

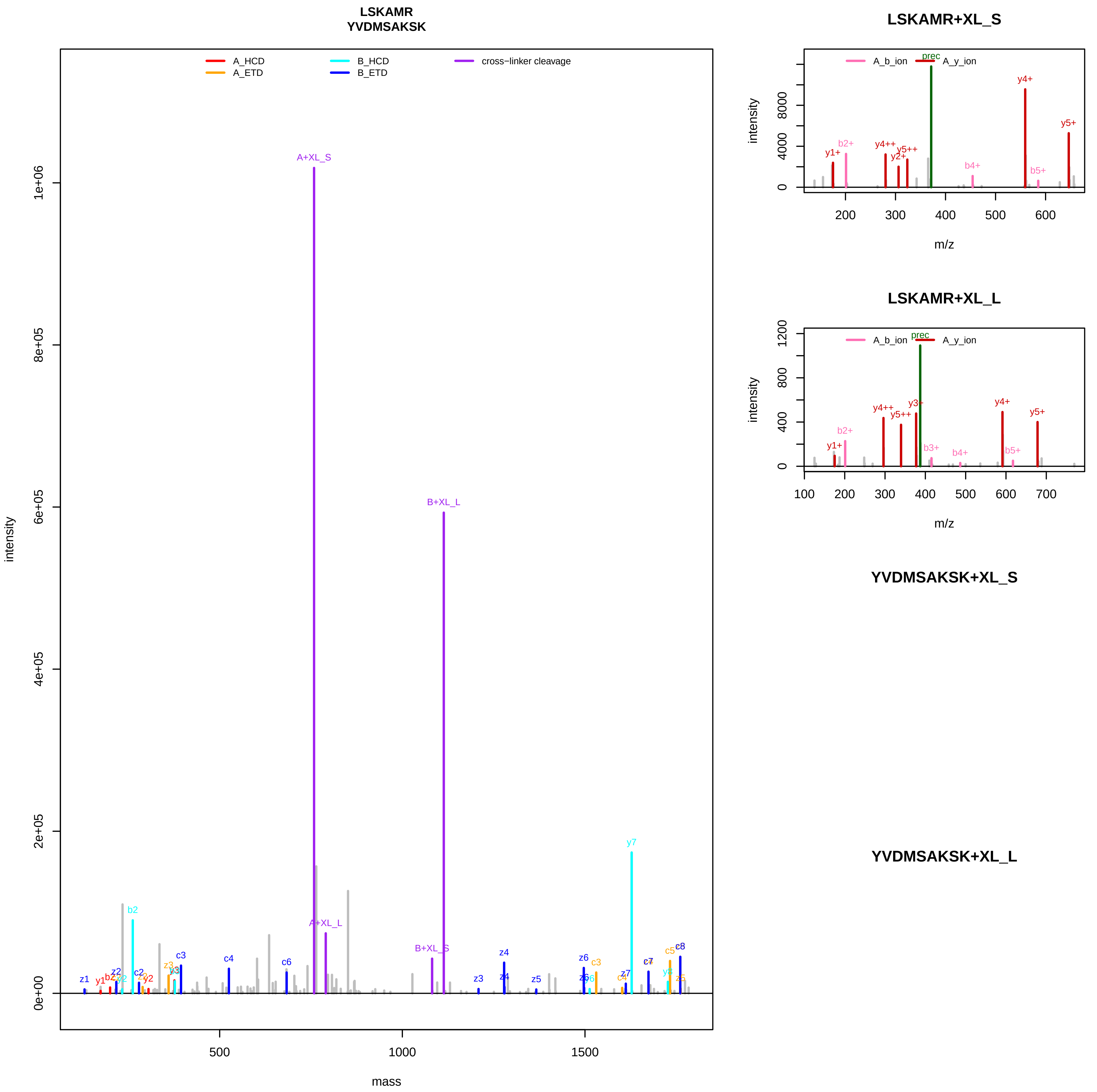
LSKAMR
YVDMSAKSK
A_HCD
A_ETD
B_HCD
B_ETD
cross−linker cleavage
A+XL_S
B+XL_L
A+XL_L
B+XL_S
y7
b2
c3
c4
c6
z4
z3
z5
z6
c7
c8
c5
intensity
mass
LSKAMR+XL_S
A_b_ion
A_y_ion
prec
y1+
b2+
y4++
y2+
y5++
b4+
y4+
b5+
y5+
m/z
LSKAMR+XL_L
y3+
b3+
YVDMSAKSK+XL_S
YVDMSAKSK+XL_L
